# A Critical Role for HlgA in *Staphylococcus aureus* Pathogenesis Revealed by A Switch in the SaeRS Two-Component Regulatory System

**DOI:** 10.3390/toxins10090377

**Published:** 2018-09-18

**Authors:** Arundhathi Venkatasubramaniam, Tulasikumari Kanipakala, Nader Ganjbaksh, Rana Mehr, Ipsita Mukherjee, Subramaniam Krishnan, Taeok Bae, M. Javad Aman, Rajan P. Adhikari

**Affiliations:** 1 Integrated Biotherapeutics Inc., Rockville, MD 20850, USA; Arundhathi@integratedbiotherapeutics.com (A.V.); Tula@integratedbiotherapeutics.com (T.K.); Nader@integratedbiotherapeutics.com (N.G.); ranamehr@gmail.com (R.M.); imukher@integratedbiotherapeutics.com (I.M.); subramaniamkrish@gmail.com (S.K.); 2 Department of Microbiology and Immunology, Indiana University School of Medicine-Northwest, Gary, IN 46408, USA; tbae@iun.edu

**Keywords:** *S. aureus*, *hlgA*, *hla*, leukotoxins, Newman, SaeS, *agr*

## Abstract

Cytolytic pore-forming toxins including alpha hemolysin (Hla) and bicomponent leukotoxins play an important role in the pathogenesis of *Staphylococcus aureus*. These toxins kill the polymorphonuclear phagocytes (PMNs), disrupt epithelial and endothelial barriers, and lyse erythrocytes to provide iron for bacterial growth. The expression of these toxins is regulated by the two-component sensing systems Sae and Agr. Here, we report that a point mutation (L18P) in SaeS, the histidine kinase sensor of the Sae system, renders the *S. aureus* Newman hemolytic activity fully independent of Hla and drastically increases the PMN lytic activity. Furthermore, this Hla-independent activity, unlike Hla itself, can lyse human erythrocytes. The Hla-independent activity towards human erythrocytes was also evident in USA300, however, under strict *agr* control. Gene knockout studies revealed that this Hla-independent Sae-regulated activity was entirely dependent on gamma hemolysin A subunit (HlgA). In contrast, hemolytic activity of Newman towards human erythrocytes from HlgAB resistant donors was completely dependent on *agr*. The culture supernatant from Newman *S. aureus* could be neutralized by antisera against two vaccine candidates based on LukS and LukF subunits of Panton-Valentine leukocidin but not by an anti-Hla neutralizing antibody. These findings display the complex involvement of Sae and Agr systems in regulating the virulence of *S. aureus* and have important implications for vaccine and immunotherapeutics development for *S. aureus* disease in humans.

## 1. Introduction

*Staphylococcus aureus* (*S. aureus*) is an opportunistic human pathogen that can cause a variety of acute and chronic infections [1,2,3,4,5]. Because of its high adaptability and genomic plasticity, *S. aureus* has been able to acquire resistance to commonly prescribed antibiotics [6,7,8], and has already emerged as a multi drug resistant “superbug”. In addition to the antibiotic treatments, therapeutic options including active immunization and passive immunotherapy have been applied, but neither of these methods has been successful. Vaccine approaches that target surface antigens of *S. aureus*, not only failed in clinical trials, but also appear to exacerbate the fatal sequelae of the disease [9]. This concern has been further reinforced in animal studies using cell-associated antigens [10,11]. Toxin-based vaccines may represent an effective alternative.

In *S. aureus*, complex networks of transcriptional regulators, two-component systems (TCSs) [12,13] and quorum sensing systems [14,15] tightly regulate numerous virulence factors including surface proteins and toxins. Three groups of pore-forming cytotoxins appear to be critical for *S. aureus* pathogenesis. The first group, called alpha toxin, or alpha hemolysin (Hla), is a single component toxin that binds to a specific cell receptor, a zinc-dependent metalloprotease called ADAM10, and forms transmembrane pores [16,17]. The second group is bicomponent pore-forming toxins (BCPFTs) that require two components (S and F), as well as specific receptors to oligomerize and form functional pores in key immune cells including polymorphonuclear cells (PMNs) [18,19,20]. The third group of toxins is small amphiphilic peptides called phenol soluble modulins (PSM) [19,21] that directly insert into the host cell membrane to form pores [21]. The PMN-lytic activity of *S. aureus* serves the purpose of immune evasion while the hemolytic activity provides the bacteria with a source of the critical nutrient-iron. 

The expression of these toxins is regulated by the *S. aureus* exoprotein expression (*Sae*) locus, a well-characterized TCS that acts as an activator of toxin production [22] along with other regulators like *agr* [15]. Several studies showed that *sae* is essential for bacterial virulence in animal models [23,24,25]. For example, the *sae* locus regulates the *hla* expression in a rabbit infective endocarditis model in the absence of both *agr* and *sarA* [26]. The *sae* locus consists of four open reading frames (ORFs): P, Q, R, and S [27,28]. The TCS consists of SaeRS [23,29], with SaeS being a transmembrane histidine kinase and SaeR, a response regulator [23]. Initially, SaePQ transcripts have been predicted to play a role as autoinducers in activation of the *saeRS* locus [27,28]; in more recent studies, the SaePQ protein complex has been shown to activate SaeS phosphatase activity [30].

SaeS senses the environmental signals through unknown ligands. The extracellular (EC) loop of SaeS plays a critical role in *S. aureus* virulence. It is the overall conformation, not the individual amino acid sequence, that is important for the function of SaeS [31,32]. The M31A mutation in this loop leads to a significant reduction in the human neutrophil cytotoxic activity in culture supernatant, whereas mutations in the two aromatic anchor residues, W32A and F33A, disrupt basal signaling of SaeS. Previously, we and other [33] reported a single amino acid mutation in the Newman SaeS protein (L18P) [27] that causes a temporal change in the regulatory network of the Newman strain. One such example is depicted by the accelerated kinetics of surface protein expression in Newman compared to other strains. In addition to the Newman strain, this SaeS-based unique expression pattern has also been reported in ST30 (CC30)-SCCmec IV (USA1100) [34].

In this report, we explored the impact of this mutation (L18P) in the Newman strain. We found that supernatant collected from the Newman strain can lyse rabbit and human red blood cells (RBC) in a Hla-independent manner. We demonstrate that this activity is dependent on gamma hemolysin A subunit (HlgA). HlgA expression is highly elevated in Newman in an Agr-independent but SaeS_L18P_-dependent manner. Furthermore, we demonstrate that the enhanced cytolytic activity and specific lysis of human RBC vary from donor to donor. HlgA specific lytic activities were more significant in blood from sensitive donors. Lysis of blood from resistant donors was independent of hla and leukotoxins, but dependent on some other proteins activated by *agr*. In this study, we have established that HlgA is a valid virulence factor and plays a role for the non-canonical pairing of leukotoxins in the pathogenesis of *S. aureus* strains.

## 2. Results

**Figure 1:**
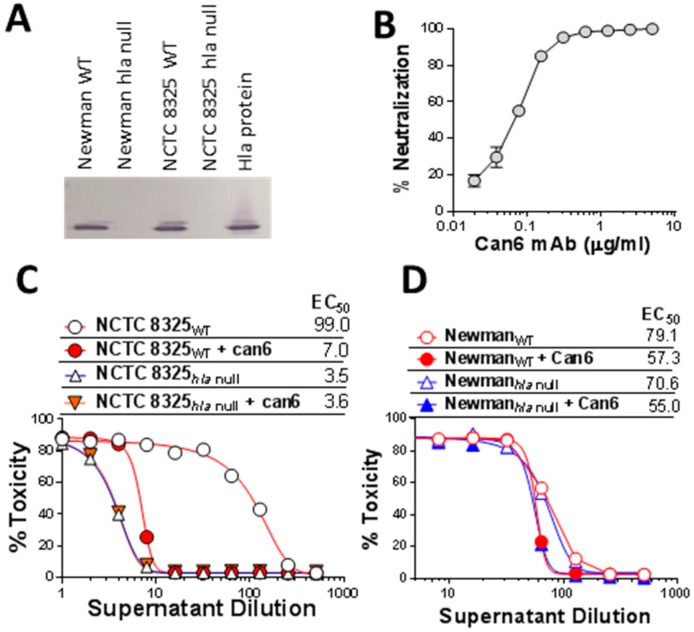
Expression pattern of Hla in Newman and NCTC8325 *S. aureus* strains and role of Hla in hemolytic activity. (**A**) Western blot analysis of supernatant from overnight culture from NCTC 8325 and Newman wild type (WT) and hla null mutants; (**B**) Neutralization of purified Hla by the monoclonal antibody Can6 in rabbit RBC assay; (**C**,**D**) Hemolytic activity of supernatants of WT and hla null mutant of NCTC 8325 (**C**) and Newman (**D**) strains in rabbit RBC assay. EC_50_: 50% toxicity titer.

The Newman strain is one of the most widely used standard laboratory strain for alpha toxin vaccine studies as well as for research on staphylococcal biology [35,36]. While evaluating a panel of anti-Hla neutralizing antibodies we noted that hemolytic activity of supernatants from the Newman culture cannot be neutralized by our anti-Hla monoclonal antibodies or even by polyclonal anti-Hla IgG, although Newman is known to express Hla [37]. This observation prompted us to examine the nature of Newman hemolytic activity. We first generated *hla* null mutants of Newman as well as NCTC 8325 as a reference strain. Western blot analysis confirmed comparable expression of Hla in both parental strains and the loss of Hla expression in the mutants (Figure 1A). We used one of our anti-Hla monoclonal neutralizing antibodies, Can6 (CAN24G4-1) [38], to characterize the hemolytic activity in these strains. In the rabbit RBC (RRBC) hemolytic assay, Can6 exhibited potent neutralizing activity towards purified Hla (Figure 1B) (IC_50_ < 1 nM) as well as the hemolytic activity of NCTC 8325 overnight culture supernatants but not NCTC 8325_*hla* null_ (Figure 1C). In contrast, in Newman background, there was no difference in hemolytic activities in the wild type versus Hla null strains and Can6 could not neutralize the hemolytic activities (Figure 1D).

### 2.1. Role of SaeS in the Newman Phenotype

**Figure 2:**
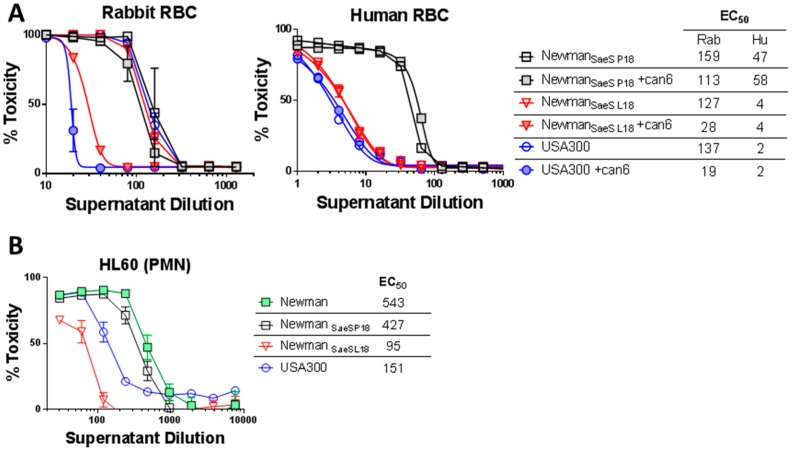
The impact of L18 mutation in SaeS on hemolytic and PMN lytic activity. (**A**) Hemolytic assays in rabbit (left panel) and human RBC (right panel) were carried out with dilutions of supernatants from Newman_Saes P18_, Newman_Saes L18_, and USA300 in presence or absence of 5 µg/mL of Can6; (**B**) Toxicity of the culture supernatants of Newman, Newman_SaeS P18_, Newman_SaeS L18_, and USA300 in HL-60 based cytotoxic assays. EC_50_ values are indicated.

We have previously reported that a point mutation in Newman SaeS (L18P) [27] results in early expression of exotoxins. This mutation has also been linked to other regulatory phenotypes [39,40]. We hypothesized that the SaeS_L18P_ mutation can be responsible for the Hla-independent hemolysis phenotype in Newman. To this end, we generated Newman *sae* mutants that were either complemented in trans with *saeS* from the Newman strain (Newman_SaeS-P18_, resembling wild type Newman) or from the USA300 strain (Newman_SaeS-L18_) and analyzed these strains for hemolytic and PMN-lytic activity as well as expression pattern of Hla and various BCPFTs. The hemolytic activity against rabbit RBC was similar in Newman_SaeS-L18_ or USA300 compared to Newman_SaeS-P18_ (Figure 2A left panel). Moreover, Can6 could only neutralize supernatants from the USA300 and Newman_SaeS-L18_ (having USA300 type *sae*). More dramatic effect of *sae* mutation became apparent when we tested human RBC lysis. While culture supernatants from USA300 or Newman_SaeS-L18_ poorly lysed human RBC, Newman_SaeS-P18_ supernatants displayed 10-fold higher hemolysis (Figure 2A right panel). This human RBC hemolytic activity was entirely related to a non-Hla component, since Can6 showed no neutralizing activity for any of the strains, consistent with previous reports that Hla does not lyse human RBC [41,42] also consistent with lack of Hla receptor ADAM10 on human erythrocytes [16,17]. Using a PMN-lysis assay we further demonstrated that the *saeS* mutation in Newman leads to approximately 3 and 5-fold higher toxicity towards PMN compared to USA300 or Newman carrying L18 *SaeS*, respectively (Figure 2B). While Newman exhibited higher lytic activity toward PMNs than USA300, reversal of the SaeS to the USA300 type largely abrogated this activity to levels much less than USA300, likely due to expression of Panton-Valentine leukocidin (PVL) in USA300 that is not expressed by Newman. Taken together, these data indicate that a single point mutation in the SaeS leads to a largely Hla-independent rabbit RBC hemolytic phenotype and increased lytic activity towards human RBC and PMNs in Newman. Reversion of this mutation makes the Newman hemolytic profile nearly indistinguishable from USA300.

### 2.2. Critical Role of Newman SaeS Mutation for In Vivo Virulence

**Figure 3:**
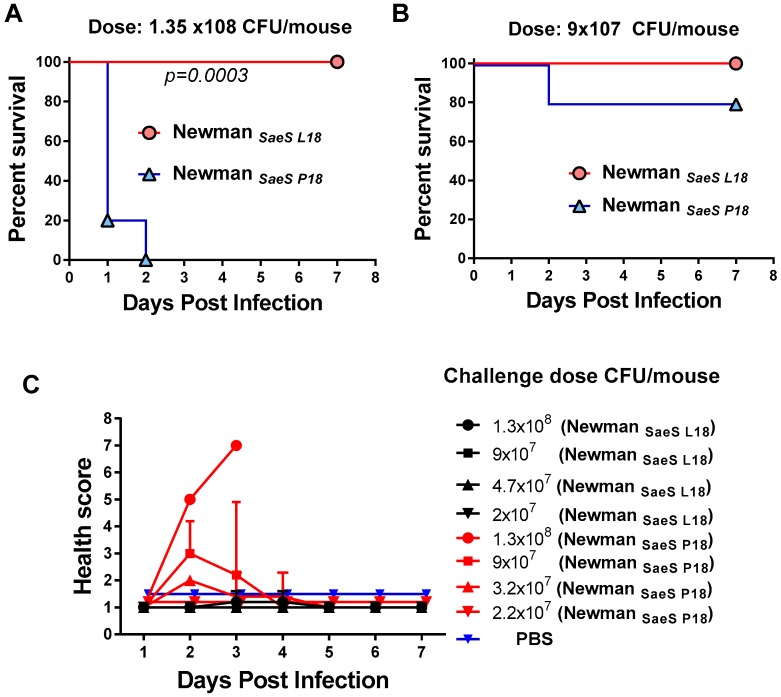
Pathogenicity of *S. aureus* Newman_Saes P18_ and Newman_SaeS L18_ pneumonia infection models. (**A**) Survival percentages of mice were compared after intranasal (IN) challenge with 1.35 × 10^8^ CFU or (**B**) 9 × 10^7^ CFU of Newman_SaeS P18_ and Newman_SaeS L18_; (**C**) Health scores were compared with four different IN challenge dose in Newman_SaeS P18_ with Newman_SaeS L18_. Statistical analysis was performed using Log-Rank (Mantel-Cox) test.

Since there was a significant difference in in vitro toxicity between Newman_SaeS-P18_ and Newman_SaeS-L18_, we sought to examine if this translates into higher virulence in a mouse pneumonia model. Outbred (CD-1) mice were better suited for this study because they are more resistant to *S. aureus* infection than Balb/C [43]. Four groups of mice (5 mice/group) were challenged with four different doses (1.3 × 10^8^ to 2 ×10^7^ CFU/mouse) of either Newman_SaeS-P18_ or Newman_SaeS-L18_. All mice challenged with 1.3 × 10^8^/mouse Newman_SaeS-P18_ died within the first two days of infection, whereas all mice infected with Newman_SaeS-L18_ survived (Figure 3A). Statistical analysis with Log-Rank (Mantel-Cox) test showed that Newman_SaeS-P18_ were significantly more pathogenic than Newman_SaeS-L18_ (*p* = 0.0003). In the lower dose group (9 × 10^7^/mouse), 80% of the mice survived in the Newman_SaeS-P18_ group whereas 100% mice survived in the Newman_SaeS-L18_ group (Figure 3B) and no lethality was observed in the lower doses of either strain. Survival data were consistent with the health score data showing that only Newman_SaeS-P18_-challenged mice were sick in all different doses tested, whereas mice in Newman_SaeS-L18_ were healthy (Figure 3C).

### 2.3. HlgA Plays A Major Role in Newman Virulence

**Figure 4:**
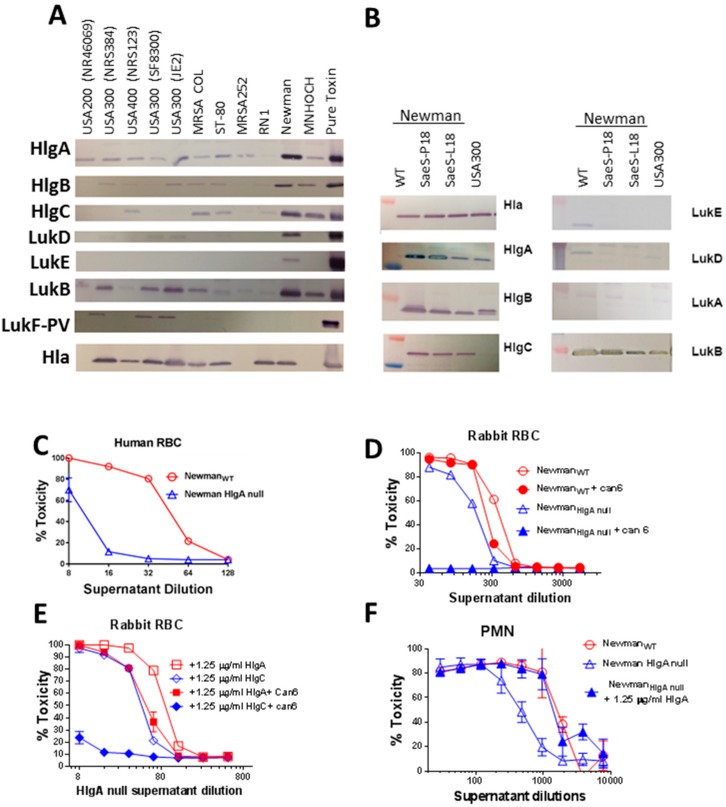
Expression pattern of Hla and leukotoxins in various *S. aureus* strains and role of HlgA in Newman phenotype. (**A**) Leukotoxin subunit expression was analyzed by Western blot in the indicated *S. aureus* strains. Respective purified toxin subunits (Lane 12) were used as positive control; (**B**) Western blots analysis of culture supernatants from Newman_SaeS-P18_, Newman_SaeS L18_, and USA300 with leukotoxin: Specific peptide polyclonal antibodies; (**C**) Toxicity of the supernatant from Newman wild types (WT) and HlgA null Newman on human RBC; (**D**) Hemolytic activity of supernatants from WT and HlgA null mutants of Newman in presence or absence of Can6 on rabbit RBC; (**E**) Supplementation of Newman_HlgA null_ with HlgA but not HlgC restore the Newman hemolytic phenotype on rabbit RBC; (**F**) Addition of exogenous HlgA increases PMN lytic activity of Newman_HlgA null_ supernatants to the level of WT Newman.

The above data clearly showed that the distinct regulatory networks of Newman_SaeS-P18_ and Newman_SaeS-L18_ lead to altered virulence in vitro and in vivo. To examine the toxin profile resulting from this single point mutation in the SaeS protein, we first tested the expression of several leukotoxins and Hla in Newman and several other *S. aureus* strains. As shown in Figure 4A, the Newman strain expressed Hla and all leukotoxins tested except for LukF-PV, with HlgA and LukB expression being particularly high. The high-level expression of HlgA was striking since HlgAB is known to be a potent toxin towards human and murine erythrocyte through binding to Duffy antigen receptor (DARC) [44]. Thus, we examined if the SaeS-P18 mutation leads to altered expression of leukocidins. As shown in Figure 4B, in comparison to wild type and SaeS-P18 reconstructed mutants, HlgA expression was repressed in Newman_SaeS-L18_ to levels similar to USA300. Expression of LukB was also reduced in the presence of SaeS-L18. In contrast, expression of Hla, HlgC, and HlgB (Figure 4B) as well as LukA and LukD was independent of L18P mutation in Newman (Figure 4B).

To examine if the increased HlgA expression was responsible for the Newman phenotype, we moved the *hlgA* transposon mutation from the JE2 strain to the Newman strain. The Newman *hlgA* mutant displayed severely reduced hemolytic activity towards human RBC (Figure 4C). The hemolytic activity of Newman_*hlgA* null_ supernatant towards rabbit RBC was reduced by approximately 50% when compared to the wild type strain (hemolysis effective concentration (EC_50_) as 350 for wild-type and 180 for mutant) (Figure 4D). However, Newman_*hlgA* null_ retained significant activity against rabbit RBC (Figure 4D). Interestingly while the hemolytic activity in wild-type Newman was marginally neutralized by Can6, this antibody completely abolished the hemolytic activity of the *hlgA* null mutant (Figure 4D). These data indicate that in the presence of HlgA overexpression, the HlgA-mediated hemolysis is dominant while, in the absence of HlgA, Newman relies on Hla for hemolysis, suggesting that the elevated expression of HlgA determines the highly virulent phenotype of Newman. To further confirm this hypothesis, we complemented Newman *hlgA* null mutant culture supernatant with 1.25 μg/mL of purified HlgA or HlgC protein, both S subunits of gamma hemolysin. Supplementing Newman_*hlgA* null_ supernatant with HlgC showed no significant effect on its hemolytic activity, while supplementing with HlgA increased the toxicity by two-fold (Figure 4E). A similar effect was observed in HL-60 based PMN assay where *hlgA* mutant had lower toxicity, and addition of HlgA protein increased its toxicity to WT levels (Figure 4F). We also supplemented this mutant supernatant with other subunits HlgB, LukE, LukD, LukS-PV, and LukF-PV concentration; however, none of these subunits complemented *hlgA* mutant phenotypes (data not shown).

**Figure 5:**
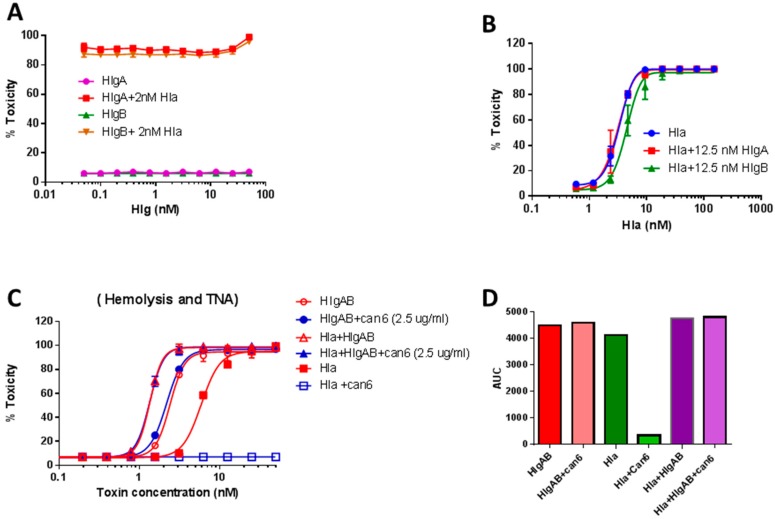
Dose response of Hla and HlgA + HlgB toxicity in 4% rabbit RBC. (**A**) Titration curve of HlgA and HlgB in the presence of 2 nM Hla; (**B**) Titration of Hla in the presence of 12.5 nM HlgA or HlgB; (**C**) 2.5 μg/mL Can6 can neutralize Hla, whereas when hla is mixed with HlgAB, Can6 cannot neutralize toxicity; (**D**) Area under the curve (AUC) based on plot (**C**).

We next used purified toxins to further examine the potential dominance of HlgAB over Hla in rabbit RBC assay. We first examined if HlgA or HlgB compete with Hla in rabbit RBC assay and observed that HlgA or HlgB alone had no impact on dose dependent lysis of rabbit RBC by Hla (Figure 5A,B) suggesting that there is no competition for receptor binding. However, consistent with the above observations, in the presence of HlgAB functional toxin Hla could only display marginal hemolytic activity as shown by the inability of Can-6 to reduce hemolysis in the presence of Hla + HlgAB (Figure 5C,D).

### 2.4. Agr Independent Lysis of Rabbit RBC in Newman

**Figure 6:**
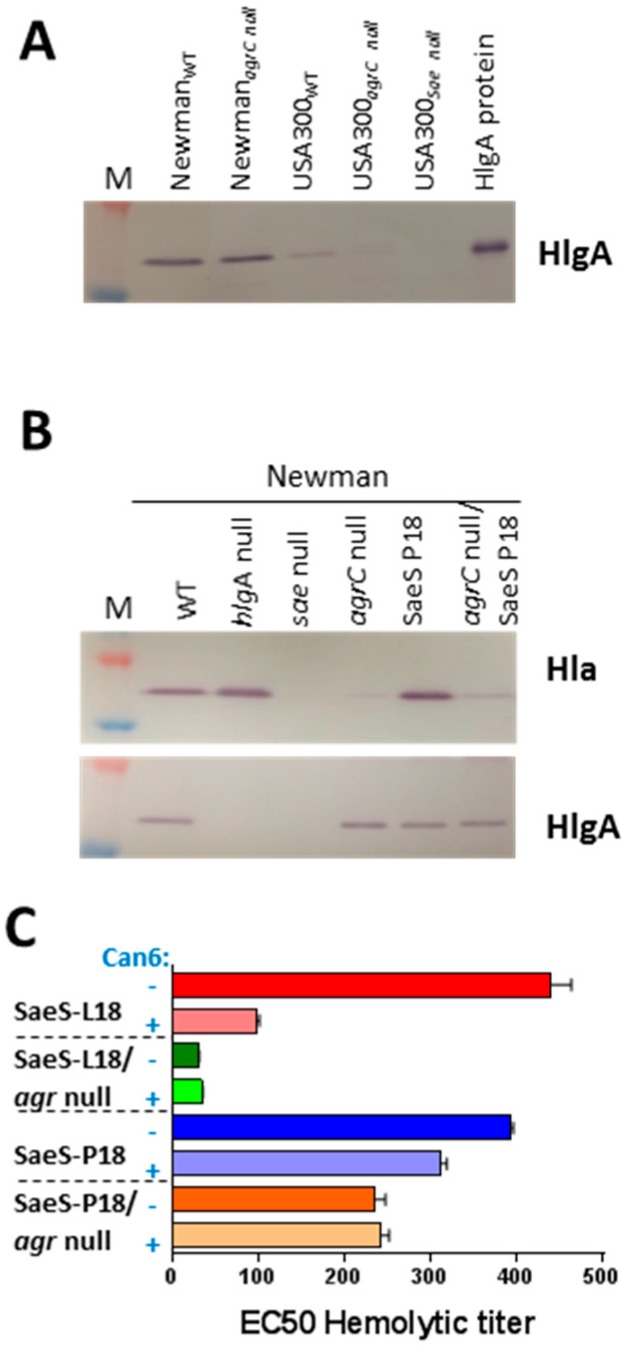
*agr*-independent regulation of HlgA subunit in the Newman strain. (**A**) Western blots were carried out with anti-HlgA polyclonal antibody in culture supernatant from *agrC* null USA300 and Newman; (**B**) Western blot analysis of Newman_SaeS L18_ and its *agrC* null mutant with anti-HlgA specific polyclonal antibody along with Hla specific monoclonal antibody; (**C**) Hemolytic and TNA titers were carried out with Newman_SaeS P18_ and its *agrC* null mutant in the presence and absence of anti-Hla mAb Can6.

RNAIII, the regulatory RNA encoded by the *agr* locus, is known as the major positive regulator of exotoxin production in *S. aureus* [15] and *hlgA* is one such gene activated by RNAIII [45]. To evaluate the role of Agr in the unique regulation of toxin production in the Newman strain, we moved an *agrC* null mutation from the JE2 background into the Newman and USA300 strains. HlgA expression was unaffected in the Newman_*agrC* null_ strain, whereas it was abrogated in both *agr* null and *sae* null mutants of USA300 (Figure 6A). These data further indicate a profound difference in the regulatory network controlling HlgA expression in Newman and USA300. We also analyzed the expression of Hla and HlgA in *hlgA* null, *agrC* null, *sae* null, WT as well as reconstituted Newman_SaeS-P18_ background. Consistent with the above data, the expression of HlgA was not affected in Newman_SaeS-P18_ *agrC* null mutant, whereas Hla expression was entirely dependent on both Agr and Sae, further confirming that the Newman version of SaeS (Newman_SaeS-P18_) specifically affects HlgA but not Hla (Figure 6B). The functional hemolytic phenotype of these mutants was consistent with the expression pattern, with the hemolytic activity in the Sae-L18 background (USA300 type) being Agr-dependent and in the Sae-P18 background (Newman type), being Agr-independent as tested in 4% rabbit RBC (Figure 6C).

### 2.5. Differential Role of Agr in Lysis of Human Erythrocytes

**Figure 7:**
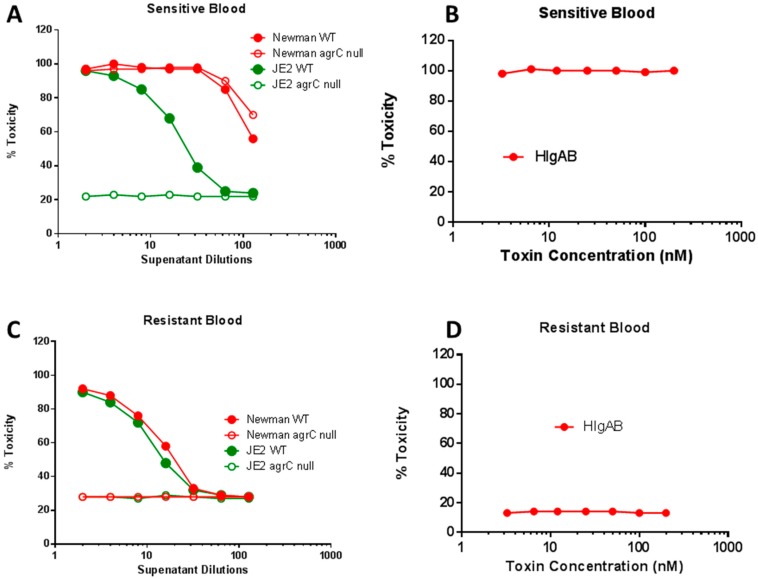
*agr* effect in lysis of sensitive and resistant human red blood cells by culture supernatant from Newman and JE2 strain of *S. aureus* versus purified HlgAB. Blood from sensitive (**A**,**B**) and resistant donor (**C**,**D**).

We have observed low hemolytic activity in *agr*-defective clinical mutants like MRSA252 (data not shown) towards human RBC. We examined the role of *agr* in human RBC lysis using USA300 (JE2) and its *agr* mutant. Human RBC lytic activity was clearly detectable in JE2, and this activity was completely abrogated in the *agr* null mutant of JE2, indicating that this activity is under full *agr* control (Figure 7A). In sharp contrast, the Newman supernatant exhibited strong Human RBC lytic activity and this activity was not affected by *agr* mutation, which is consistent with our findings described above. Spann et al. previously reported that RBC from a portion of the population is resistant to lysis by HlgAB due to a polymorphism in the Duffy antigen receptor for chemokines (DARC) gene [44]. We screened RBCs from multiple individuals and identified several resistant donors. Purified HlgAB lysed sensitive blood with high hemolytic titer (Figure 7B). Interestingly, when the hemolytic activity was tested in RBC from resistant donors, the lytic activity of the both wild type JE2 and Newman exhibited similar levels of lysis, whereas *agr* null in both backgrounds completely abrogated the lytic activity (Figure 7C) further indicating that the *agr*-dependent activity can only manifest itself in the absence of HlgAB functionality. As expected, the resistant blood could not be lysed by purified HlgAB (Figure 7D).

### 2.6. Lysis of Human RBC Requires Free N-Terminus of HlgB

**Figure 8:**
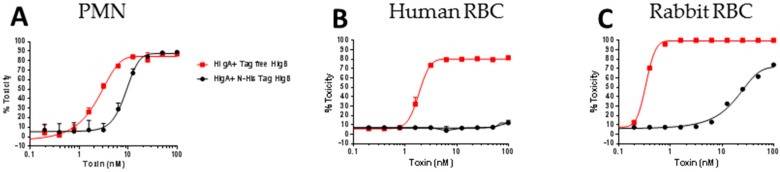
Cytotoxicity of N-His tag HlgB versus tag free HlgB. (**A**) Polymorphonuclear phagocyte (PMN) lytic activity; (**B**) Hemolytic activity towards 2% human RBC; (**C**) Hemolytic activity towards 4% rabbit RBC.

In our previous study, using N-terminally His-tagged HlgA and HlgB, we had observed extremely low toxicity of His-HlgAB towards human RBC, whereas, the non-canonical tag free pair HlgA + LukD was highly hemolytic [42]. The data described above and also reported by Spann et al [44] showing full activity of non-tagged HlgAB suggested that the lack of activity of His-HlgAB was related to the N-terminus of HlgB being occupied, since His-HlgA was fully functional in combination with LukD [42]. Interestingly, while there was little difference between the PMN-lytic activity (Figure 8A) of tagged and tag-free HlgAB, N-terminally tagged HlgAB was completely inactive towards human RBC (Figure 8B) and largely attenuated toward rabbit RBC (Figure 8C). These data indicate that the N terminus of HlgB plays a critical role in lysis of human RBC.

### 2.7. Antibodies Elicited by PVL-Based Vaccines Neutralize Human RBC Hemolytic Activity

**Figure 9:**
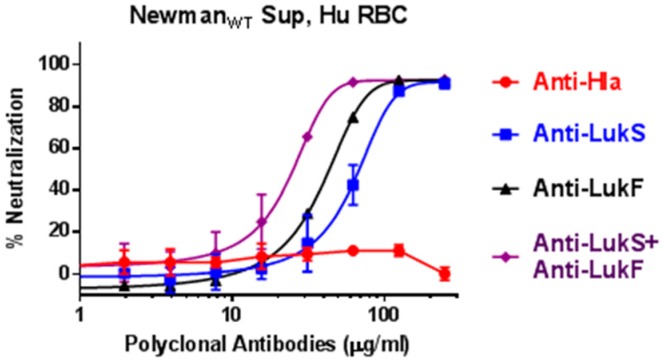
Neutralization of human RBC hemolytic activity in overnight culture supernatants of Newman_WT_ using antibodies against hla versus Panton-Valentine leukocidin (PVL) subunits.

We have previously reported two mutants of PVL subunits, LukS_mut9_ (LukS_T28F/K97A/S209A_) and LukF_mut1_ (LukF_K102A_), as promising vaccine candidates with cross-neutralizing activity against other BCPFTs [42,46]. In light of our new findings, we further tested polyclonal antibodies against LukS_mut9_, LukF_mut1_, or Hla in supernatants of Newman_WT_. As shown in Figure 9, antibodies against LukS and LukF but not anti-Hla were able to effectively neutralize the hemolytic activity. A combination of antibodies against both subtypes provided the highest level of neutralization. The effectiveness of antibodies against both subunits further indicates that the responsible toxin for the Hla-independent human RBC lysis contains both S and F subunits and further justifies the use of both subunit toxoids in a multivalent vaccine.

## 3. Discussion

The virulence of *S. aureus*, particularly the recently emerging community-acquired MRSA (CA-MRSA) clones, is largely related to its ability to produce a variety of hemolysins and leukotoxins [47,48]. *S. aureus* controls the expression of many of these secreted toxins using the TCS environmental sensing systems *agr* and *sae*. Alpha hemolysin (Hla), a toxin regulated by both *agr* and *sae* [26], is known as the primary mediator of red blood cell lysis providing a rich source of iron for bacterial growth [49]. Bicomponent leukotoxins including PVL, HlgAB, HlgCB, LukED, and LukAB (also known as LukGH) are primarily known for their lytic activity towards the polymorphonuclear phagocytes (PMNs) [50], while HlgAB and LukED also possess hemolytic activity [44]. Here, we demonstrate that the Sae-regulated HlgA production conveys remarkably high virulence to *S. aureus* and provide evidence that this increased virulence is correlated with in-vivo and in-vitro studies.

The *S. aureus sae* locus encodes the environmental sensor histidine kinase SaeS and the response regulator SaeR that constitute the two-component system as well as two additional proteins SaeP and Q [28]. The N-terminus of SaeS consists of two transmembrane (TM) domains flanking a 9-residue extracellular loop. A recent study showed that kinase activity of SaeS is determined by the extracellular loop, as mutating this linker leads to elevated kinase activity even in the absence of any extracellular signals [32]. It is believed that the TM domains and the extracellular loop control the kinase activity of SaeS via conformational changes [32]. Previously we reported that a single point mutation within the first TM domain of SaeS (L18P) in the *S. aureus* strain Newman protein results in expression of multiple exoproteins in the earlier log phase compared to other WT strains [27]. The L18P mutation introduces a kink in the middle of the TM1 alpha helix, likely inducing conformational changes in the SaeS histidine kinase leading to its augmented activity. This is consistent with previous reports of a constitutively active status of *sae* TCS in Newman [33,51]. Here, we report that this mutation creates a regulatory switch rendering most of the cytolytic activity as well as in vivo pathogenesis of the Newman strain dependent on HlgA.

Our analysis of the L18P mutation further revealed a dominant Hla-independent hemolytic activity in *S. aureus* Newman. We noted that despite the high level of Hla expression, more than 90% of the hemolytic activity in the Newman supernatant remained non-neutralized by an anti-Hla neutralizing antibody. Consistent with this, Newman_*hla* null_ retained nearly all of its hemolytic activity towards rabbit RBC, while the *hla* deletion largely abrogated this activity in NCTC 8325. This Hla-independent hemolytic phenotype was entirely reversed by mutating the proline at position 18 in Newman SaeS back to leucine. In contrast to Newman, the hemolytic activity of USA300 (NRS384) carrying L18 SaeS towards rabbit RBC was entirely Hla-dependent. The L18P mutation also dramatically increased the PMN-lytic activity of Newman as compared to USA300. We further demonstrate that this phenotype is responsible for increased in vivo virulence in a mouse model of *S. aureus* pneumonia. At doses as high as 1.3 × 10^8^ CFU, replacing the Newman *SaeS* with that of USA300 completely abrogated virulence both in terms of lethality and weight loss underscoring the critical role of this single mutation. These findings provide a molecular explanation for the previously reported highly virulent nature of *S. aureus* Newman [35,36,52,53,54].

The Hla-independent hemolytic phenotype of Newman was entirely due to upregulation of HlgA. Deleting the *hlgA* gene reverted this phenotype to a fully Hla-dependent hemolysis indicating that HlgA dominated the hemolytic activity when co-expressed with Hla. Furthermore, supplementing the Newman_*hlgA* null_ supernatant with purified HlgA restored the Hla-independent phenotype. This Sae-dependent HlgA expression in Newman was fully independent of *agr*, while HlgA expression in USA300 required both *Sae* and *Agr*. These data are consistent with the constitutive activity of Sae in Newman being independent of *agr* [51]. An interesting aspect of our findings is the interplay between Hla and HlgAB and Agr versus Sae dependent activities. Our findings show that in Newman while both toxins are present the Sae-dependent HlgAB activity is dominant. This was corroborated by in vitro experiments using purified toxins (Figure 5). While neither HlgA or HlgB subunits alone had any impact on Hla-mediated lysis of rabbit RBCs, in presence of HlgAB, the lysis was entirely dependent on HlgAB. We further show that, in blood from human donors that are sensitive to HlgAB, lysis of RBCs by Newman is fully independent of *agr*, while the same activity in USA300 is completely abrogated by mutating *agr*. In contrast, when blood from HlgAB resistant donors was used, both Newman and USA300 activities were entirely dependent on *agr*. Since Hla cannot lyse human RBC, the nature of this *agr*-dependent human RBC lytic activity as well as the basis of this dominance remains to be determined.

Within the infected hosts, bacteria are under enormous stress because they constantly encounter antimicrobial peptides and reactive oxygen species produced by immune cells as well as an environment low in key nutrients such as iron. Iron is an essential nutrient for all living organisms, and bacterial pathogens have evolved a variety of mechanisms to scavenge iron from the high-affinity iron-binding proteins and transport them into the cells. *S. aureus* is strictly dependent on acquiring iron in the host for its survival and persistence within abscesses [55,56,57,58,59]. In vertebrates, 99% of the iron is located intracellularly [60] and mostly stored in erythrocytes in complex with hemoglobin. Since the intracellular pool of iron is not available to bacterial pathogens, many bacteria including *S. aureus* produce hemolytic toxins to lyse the erythrocyte and release iron for their growth. Electron microscopy studies have demonstrated the abundance of erythrocytes within *S. aureus* abscess providing an immediate source of iron for bacterial growth. *S. aureus* may have gained the *sae* mutation to acquire growth advantage by more effectively lysing human RBCs.

Currently toxoid based vaccines for *S. aureus* are receiving renewed attention. Our findings of the importance of HlgAB suggest that an effective toxoid vaccine must be also able to neutralize this toxin. HlgA and HlgB have a high degree of sequence identity with LukS-PV and LukF-PV, respectively [18,19,20]. Our findings show that polyclonal antibodies against two toxoids of LukS-PV and LukF-PV can block the hemolysis of human erythrocytes by supernatants of Newman WT *S. aureus* while anti-Hla antibody had no effect. This finding has important implications for treatment and vaccination against *S. aureus*, as vaccines and immunotherapy neutralizing HlgA alone could be developed that severely hamper the ability of *S. aureus* to access iron during infection in humans.

## 4. Materials and Methods

### 4.1. Growth Media and Bacterial Strains

General method applied for bacterial culture was described in previous literature [27,61]. *S. aureus* strains were grown in brain heart infusion broth/agar (BHI) media at 37 °C overnight. Transduction and selection of transductants were carried out by standard transduction techniques with phage 80α [27]. Most of the bacterial mutants used in this paper were obtained from the NARSA (now BEI) repository. Strains used in this study are listed in the following table (Table 1).

### 4.2. Western Blot Analysis

Unique peptide specific rabbit polyclonal antibodies were generated targeting unique sequences of HlgABC, LukED, LukAB and LukF (from GenScript) to specifically detect these proteins. SDS-PAGE and Western blots were carried out using iBlot 2 Dry Blotting System (from ThermoFisher Scientific) as per manufacturer’s instructions.

**Hemolytic and toxin neutralization assays:** Hemolytic assays were performed based on our previously published report [42], with RBC from Rabbit that were purchased from Colorado Serum company, Co. (Denver, Colorado, USA) and human blood that was purchased from BIoreclamation IVT Inc. (Baltimore, MD, USA). For Rabbit RBC assays, blood was used within 10 days of the date of blood drawn because the hemolytic titer from older blood samples falls out of specification. Human blood was used within 1 month. Whole blood from both human and rabbit were washed twice with PBS and re-suspended in PBS to obtain 8% (wt/vol.) for rabbit RBC and 4% (wt/vol.) for human RBC. 100 µL of blood was mixed with 100 µL of diluted culture supernatant in 96-well ELISA plates resulting in 4% and 2% of final RBC for rabbit and human, respectively. Plates were incubated at 37 °C for 30 min and centrifuged for 5 min at 3.5 K RPM in Sorvall. 100 μL of supernatants were transferred to NUNC ELISA plates, without disturbing the pellet. OD 416 nm was read, and 4-PL plots were generated with diluted supernatant by using Softmax (Molecular devices). EC_50_ values (hemolytic titers) were calculated and defined as supernatant dilution at which 50% of hemolysis occurs.

Toxin neutralization assays were carried out using an anti-alpha toxin monoclonal antibody called Can6 and rabbit anti-Hla, anti-LukS and anti-LukF polyclonals (GenScript). Can6 (CAN24G4-1) is a well characterized reagent for its Hla neutralizing titer [38]. In neutralization assays, diluted culture supernatants were incubated at room temperature with antibodies for 10 min, allowing antibodies to neutralize toxins in the supernatants, followed by incubation at 37 °C.

**PMN lysis assays:** PMN cytotoxicity was determined in dimethyl sulfoxide (DMSO) induced HL-60 cells (ATCC, Manassas, VA, USA). The HL-60 cells were cultured for seven days in RPMI media supplemented with 20% fetal bovine serum (FBS) and 1.5% DMSO for optimal induction. Cell induction was confirmed by the expression of CD11b on induced versus non-induced cells using flow cytometry analysis. The cells were then harvested and washed with RPMI media containing 2% FBS. Overnight bacterial culture supernatants were normalized based on culture OD 600 nm. Supernatants were filtered through sterile 0.2 µm filter. Sterility of the supernatants were confirmed by culturing 20 μL of the filtered supernatants on BHI agar plate. Serially two-fold diluted culture supernatants were then mixed with HL-60 derived neutrophils at a final density of 5 × 10^5^ cells/well, then incubated for 3 h at 37 °C and 5% CO_2_. Cells and BHI broth alone controls were also included, and all samples were run as quadruplicates. Upon cell-supernatant incubation, this mixture was further incubated with 100 μg/mL of XTT with 1% electron coupling solution (Cell signaling) for 16 h and the cell viability was measured by colorimetric measurement at OD 470 nm (background subtracted at OD 690 nm).

**In vivo experiments:** In vivo experiments were performed for SaeS complemented Newman strains (Newman_SaeS P18_ or Newman_SaeS L18_) using CD-1 mice from Charles River that were 10–12 weeks old during the studies. Animals were housed under pathogen-free conditions and fed laboratory chow and water ad libitum. Animal studies were conducted per approved protocol by Institutional Animal Care and Use Committees (IACUC) at Nobel Life Sciences (Gaithersburg, MD, USA).

**Challenge dose preparation and back-titer:** Bacteria were streaked from glycerol stocks on BHI agar media for single colonies. From BHI plates, isolated colonies were inoculated into 50 mL Falcon tubes containing 7 mL of BHI broth. Tubes were incubated in a shaking incubator at 230 rpm at 37 °C for 16 h. Cells were harvested and washed two times with sterile PBS. The pellets were then re-suspended in required volume of PBS to make the target challenge dose. From each challenge dose, suspension aliquots were back-titered to calculate the actual challenge dose and converted to CFU/mL.

**Lethal Pneumonia model:** For the pneumonia model, isoflurane was used to anesthetize mice, followed by intranasal (IN) inoculation of a target dose of *S. aureus* Newman (Newman_SaeS P18_ or Newman_SaeS L18_) in 50 μL PBS. Animals were monitored for 7 days post challenge for morbidity (weight, and health score) and mortality two times daily until termination of study. Animal studies were conducted per approved protocol by IACUC at Nobel Life Sciences (Gaithersburg, MD, USA).

## Figures and Tables

**Table 1:** Bacterial strains used.

Strain	Genotype/Description	Source
JE2	USA300 WT from Nebraska mutant library	NARSA repository
NCTC 8325, MRSA Col, USA200, USA400, MNHOCH	Standard *S. aureus* strain	NARSA repository
MRSA ST-80	Standard *S. aureus* strain	From Dr. Jean Lee, BWH, Harvard University
USA300 (NRS384)	Community MRSA strain	NARSA repository
Newman	MSSA strain	[61]
NE1399	JE2 (hlgA null)	NARSA repository
NE1354	JE2 (Hla null)	NARSA repository
NE873	JE2 (AgrC null)	NARSA repository
Newman_SaeS P18_	Newman with SaeS version from Newman	From Dr. Bae
*agrC* Newman_SaeS P18_	80α transductant of NE873 into Newman_SaeS P18_	This work
Newman_SaeS L18_	Newman with SaeS version from USA300	From Dr. Bae
*agrC* Newman_SaeS L18_	80α transductant of NE873 into Newman_SaeS L18_	This work
*agrC* null Newman	80α transductant of NE873 into Newman	This work
*agrC* null USA300 NRS384	80α transductant of NE873 into USA300	This work
*hlgA* null Newman	80α transductant of NE1399 into Newman	This work
*hla* null Newman	80α transductant of NE1354 into Newman	This work
